# Asymptomatic Dengue Virus Infections, Cambodia, 2012–2013

**DOI:** 10.3201/eid2507.181794

**Published:** 2019-07

**Authors:** Sowath Ly, Camille Fortas, Veasna Duong, Tarik Benmarhnia, Anavaj Sakuntabhai, Richard Paul, Rekol Huy, Sopheak Sorn, Kunthy Nguon, Siam Chan, Souv Kimsan, Sivuth Ong, Kim Srorn Kim, Sowathy Buoy, Lim Voeung, Philippe Dussart, Philippe Buchy, Arnaud Tarantola

**Affiliations:** Institut Pasteur du Cambodge, Phnom Penh, Cambodia (S. Ly, C. Fortas, V. Duong, S. Sorn, K. Nguon, S. Chan, S. Kimsan, S. Ong, P. Dussart, P. Buchy, A. Tarantola);; University of California, San Diego, California, USA (T. Benmarhnia);; Institut Pasteur, Paris, France (A. Sakuntabhai, R. Paul);; Malaria National Center, Phnom Penh (R. Huy);; Kampong Cham Provincial Hospital, Kampong Cham, Cambodia (K.S. Kim);; Prey Chhor District Referral Hospital, Kampong Cham (S. Buoy);; Tboung Khmum District Referral Hospital, Thoung Khmum, Cambodia (L. Voeung);

**Keywords:** dengue, asymptomatic, risk factors, epidemiology, Asia, Cambodia, Mekong, mosquitoes, viruses, vector-borne infections, dengue virus, Aedes aegypti, Aedes albopictus

## Abstract

We investigated dengue virus (DENV) and asymptomatic DENV infections in rural villages of Kampong Cham Province, Cambodia, during 2012 and 2013. We conducted perifocal investigations in and around households for 149 DENV index cases identified through hospital and village surveillance. We tested participants 0.5–30 years of age by using nonstructural 1 rapid tests and confirmed DENV infections using quantitative reverse transcription PCR or nonstructural 1–capture ELISA. We used multivariable Poisson regressions to explore links between participants’ DENV infection status and household characteristics. Of 7,960 study participants, 346 (4.4%) were infected with DENV, among whom 302 (87.3%) were <15 years of age and 225 (65.0%) were <9 years of age. We identified 26 (7.5%) participants with strictly asymptomatic DENV infection at diagnosis and during follow-up. We linked symptomatic DENV infection status to familial relationships with index cases. During the 2-year study, we saw fewer asymptomatic DENV infections than expected based on the literature.

Annually, ≈390 million people in >100 countries are infected with dengue virus (DENV); 70% of cases occur in countries in Asia (*1*). DENV is a flavivirus transmitted by *Aedes aegypti* and *Ae. albopictus* anthropophilic female mosquitoes. DENV has 4 distinct serotypes, DENV-1–4 (*2*); DENV infections can range from asymptomatic to life-threatening.

In Cambodia, the national dengue surveillance system reported 60,000 cases and 135 deaths attributed to DENV in 2012 and 2013 (*3*). Syndromic surveillance and random testing of dengue-like cases in referral pediatric hospitals in Cambodia likely underestimate the true disease burden (*4*). By definition, syndromic surveillance does not detect asymptomatic DENV infections, which increase vector transmission potential (*5*). Mammen et al. used both dengue-positive and dengue-negative index cases of febrile children to initiate perifocal investigations and found no cases in proximity to dengue-negative index cases (*6*). To maximize the number of recruited cases, we investigated homes around preidentified, dengue-positive index cases, as per a previous study (*7*). Our objectives were to document the proportion of strictly asymptomatic infections in this region of Cambodia; characterize human, sociodemographic, household-level, and mosquito control–related factors associated with DENV infection; and identify factors associated with asymptomatic DENV infection.

## Methods

### Ethics Considerations

The study protocol was approved by the Cambodian National Ethics Committee on Health Research. We obtained informed consent from participants or their guardians documented during hospital or village surveillance or perifocal investigations.

### Study Site

We conducted a study in rural villages of Kampong Cham Province, 120 km northeast of Cambodia’s capital, Phnom Penh. The study area included 368 villages with ≈60,000 households and 3 hospitals within a 30-km radius. Dengue is endemic in the region and mainly affects children <15 years of age during the annual rainy season (June–October). 

**Identification of Dengue Index Cases in Hospitals and Villages**

During June 1–October 31, 2012 and 2013, we identified DENV index cases in 3 referral hospitals and 26 villages under active surveillance for febrile illness (*5*). We targeted persons 0.5–30 years of age. In the 3 hospitals, blood samples were drawn at admission and discharge for all patients suspected of having DENV infection on the basis of clinical assessment and platelet count. In the 26 villages, volunteers monitored eligible residents weekly, measuring axillary body temperature using a digital thermometer to identify persons with temperatures >38°C. Blood samples were drawn 1–2 days after fever onset, as described elsewhere (*4*,*8*,*9*). All samples were screened for DENV infection by using a nonstructural (NS) 1 IgM/IgG combination rapid test. We confirmed DENV by using quantitative reverse transcription PCR (qRT-PCR) or NS1-capture ELISA and included case-patients with confirmed DENV infection as index cases in the study.

**Perifocal Investigations**

Within 1–2 days of identifying an index case, whether from village or hospital surveillance, we began a perifocal investigation of the index case-patient’s village of origin (*7*). For each perifocal investigation, we used a rapid dengue test kit to screen eligible residents in the index case-patient’s household for DENV and completed a baseline questionnaire on individual symptoms, socioeconomic status, and household characteristics. We did the same in 20 households in a 100-meter radius of the index case-patient’s household. We included persons 0.5–30 years of age who consented or whose guarantor consented. We tested adults >20 years of age during the first year of the study but found no DENV-positive cases and did not test this age group during the second year. All consecutive cases were eligible for inclusion. To avoid bias through overlapping investigations of a potentially common source of infection, we did not conduct a perifocal investigation within 1 week of a previous investigation for >2 index cases consecutively detected from the same village.

### DENV Testing and Case Definitions

To screen for DENV infection during surveillance and perifocal investigations, investigators tested all blood samples on-site using SD BIOLINE Dengue Duo kit (Standard Diagnostics, https://www.alere.com), according to the manufacturer’s instructions. Investigators interpreted results within 15–20 minutes and ruled out possible cases if the control band was negative. Blood samples from DENV-positive participants were sent to Institut Pasteur du Cambodge (Phnom Penh, Cambodia) for qRT-PCR testing, as described previously (*10*,*11*), or confirmation using an NS1-capture ELISA (*11*,*12*) with positive controls diluted to the limit of detection, negative, and nontemplate controls used during extraction and PCR steps to reduce inaccuracies (*10*). We considered cases confirmed when a blood sample tested positive by NS1 rapid test and was confirmed by NS1-capture ELISA or qRT-PCR. During the first year, we also tested participants for Japanese encephalitis virus (JEV) and chikungunya virus (CHIKV) IgM antibodies by ELISA and confirmed IgM-positive results using specific RT-PCR to ensure that symptoms were not related to CHIKV, JEV, or co-infections (*11*–*13*).

Symptomatic DENV-confirmed case-patients had fever, muscle or joint pain, rash, bleeding, prolonged headaches, or digestive signs. We asked participants whether they had taken antipyretics in the previous 24 hours. We termed afebrile all symptomatic DENV-positive participants without a fever and no antipyretic use. We considered participants asymptomatic when they had confirmed DENV infection, no antipyretic use, and no signs or symptoms, including fever. Participants who were symptomatic at initial diagnosis on day 0 received follow-up monitoring on days 2 and 7. We monitored asymptomatic participants daily on days 0–7 and again on day 10 using a questionnaire to document signs and symptoms of DENV. In our analyses, we recategorized participants who were asymptomatic at baseline to symptomatic if they reported any symptoms during the follow-up period.

### Statistical Analysis

We described DENV infection attack rates for perifocal investigations and the proportion of asymptomatic cases among all DENV infections and circulating serotypes. To explore participant- and household-level factors associated with DENV infection, we conducted a multivariable Poisson regression estimating attack rate ratios (ARRs) (*14*), excluding index cases. We built explanatory models around each participant-level and household-level factor, with and without adjusting for covariates. Participant-level factors included age, sex, occupation or schooling, and relationship to an index case-patient. Because we found collinearity between age and occupation, we adjusted only for age. We placed participants 0.5–1 year of age into a specific category to account for differences in immunity and exposure to vectors due to reduced mobility. Household-level factors included the main source of income, source of water, measures against mosquitoes, and environmental factors favorable to mosquito development. We further divided the source of water into 2 categories: piped water (from indoor or outdoor taps with a tube well and pump) or nonpiped water (from a pond, river, lake, or a well without pump).

Considering the limited flight range of a female *Aedes* mosquito, we assumed that the probability of DENV transmission would be higher within a household than across households. To account for this factor and measure potential clustering, we developed a random-effects multilevel model. We computed the intraclass correlation coefficient as the proportion of the variability in the probability of infection attributable to differences between households versus differences within households (*15*). We excluded 19 participants with missing covariates or predictors from the regression analyses. We explored associations between asymptomatic DENV infection and DENV serotype, participant-level factors, and the main source of income as socioeconomic indicators. We used the Fisher exact test for comparing proportions, the Student *t* test for means, and an empty multilevel model to search for a cluster effect. We conducted analyses using Stata version 13 (StataCorp, https://www.stata.com).

## Results

### Dengue Surveillance for Index Case Identification

We identified 1,294 suspected DENV-infected persons, 834 (64.5%) among hospital inpatients and 460 (35.5%) through febrile illness surveillance in villages. Our testing confirmed 555 (66.5%) DENV-positive cases among hospital patients and 36 (7.8%) DENV-positive cases through febrile illness surveillance in villages.

### Perifocal Investigations

From the 591 DENV-positive patients, we selected 149 (25.2%) consecutive cases for which we conducted perifocal investigations: 131 from hospital patients, termed PI-H, and 18 from village febrile surveillance, termed PI-V. Perifocal investigations took place in 104 villages over the 2 rainy seasons and documented 7,960 participants, 6,811 (86%) male and 1,149 (14%) female, in 2,988 households (Figure). 

**Figure:**
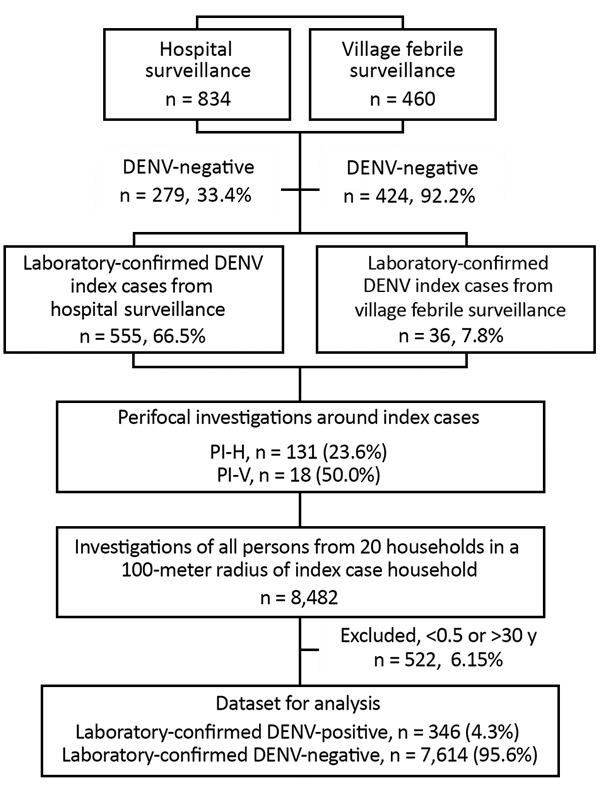
Participant screening and data flowchart for perifocal investigations for asymptomatic DENV infection, Cambodia, 2012–2013. Initial DENV screening of febrile cases was conducted using nonstructural (NS) 1 IgM/IgG combo rapid test. Perifocal investigations took place in villages of index cases; we screened all persons in 20 households within a 100-m radius of an index case household. We excluded persons <0.5 and >30 years of age. Laboratory confirmation of DENV was conducted through quantitative reverse transcription PCR or NS1-capture ELISA. DENV, dengue virus; PI-H, perifocal investigations conducted for index cases identified through hospital surveillance; PI-V, perifocal investigations conducted for index cases identified through village febrile surveillance.

We found 346 (4.3%) persons who were positive for DENV infection, 225 (65.0%) of whom were <9 years of age. We determined attack rates of 14.7/1,000 participants (14/952) in PI-V and 47.4/1,000 (332/7,008) in PI-H (p<0.05). The attack rate over the 2 outbreak seasons increased marginally from 37/1,000 persons 0.5–30 years of age during the 2012 season to 46/1,000 among those 0.5–20 years of age during 2013 (p = 0.056). Only 26 (7.5%) of 346 DENV-positive participants remained strictly asymptomatic during the 10-day follow-up, an asymptomatic DENV-infection attack rate of 3.3/1,000 over the 2 years of our study. The proportion of asymptomatic infections was 21.4% (3/14) in PI-V and 6.9% (23/332) in PI-H. 

Besides headache and fever, symptomatic case-patients mainly experienced muscle, retro-orbital, and joint pain. Although fever is considered a typical symptom of DENV infection, careful interview, rigorous clinical assessment, and follow-up interviews showed that participants remained afebrile in 110 (31.8%) of the 320 symptomatic DENV infections, even without antipyretics. Only 6 (1.7%) of the DENV-positive case-patients required hospitalization, 2 with bleeding. 

The 2 annual outbreaks were dominated by DENV-1. However, DENV-2 and DENV-4 emerged in 2013, and we detected DENV-3 sporadically (Table 1). During the first year of the study, samples from all symptomatic and asymptomatic DENV cases were negative for CHIKV by MAC-ELISA. Because we diagnosed no CHIKV in year 1, and our national surveillance system also did not detect any CHIKV cases (data not shown), we did not perform CHIKV testing during year 2. Of 26 asymptomatic cases confirmed by qRT-PCR or NS1-capture ELISA, 6 had positive JEV serology and also were positive for DENV IgM. We could not conclude whether JEV-positive results were indicative of a recent or acute JEV co-infection or the result of cross-reaction among flaviviruses. Among hospitalized patients, 2 had positive JEV results without detectable DENV IgM, even though qRT-PCR or NS1-capture ELISA was positive. These results could suggest a recent or acute JEV co-infection. During perifocal investigations, 42 participants tested positive for JEV by MAC-ELISA with negative DENV results, NS1, and qRT-PCR, supporting evidence of JEV co-circulation in the country (*16*). 

**Table 1:** Surveillance data from perifocal investigations for asymptomatic dengue virus, Cambodia, 2012–2013*

Surveillance data	2012	2013	Total
No. participants	2,391	5,569	7,960
No. villages investigated	35	77	104
No. perifocal investigations conducted	47	102	149
Mean no. participants per perifocal investigation	51	55	53
Confirmed infections, no. (%)	88	258	346
Strictly asymptomatic	5 (5.7)	21 (8.1)	26 (7.5)
Afebrile	33 (37.5)	77 (29.8)	110 (31.2)
Symptomatic	83 (94.3)	237 (91.9)	320 (92.5)
Attack rate/1,000 participants, %	36.8	46.3	43.5
Asymptomatic infections	2.1	3.8	3.3
Symptomatic infections	34.7	42.6	40.2
Afebrile infections	13.8	13.8	13.8
Symptoms at diagnosis or follow-up, no. (%)	83	237	320
Fever	55 (66.2)	180 (75.9)	236 (73.8)
Headache	52 (62.7)	169 (71.3)	221 (69.1)
Muscle pain	16 (19.3)	73 (30.8)	89 (27.8)
Retro-orbital pain	17 (20.5)	73 (30.8)	90 (28.1)
Joint pain	17 (20.5)	68 (28.7)	85 (26.5)
Rash	15 (18.1)	53 (22.4)	68 (21.3)
Any bleeding	13 (15.7)	50 (21.1)	63 (19.7)
Hospitalizations, no. (%)	3 (3.5)	8 (3.3)	11 (3.3)
DENV infections	88	258	346
Serotype, no. (%)			
DENV-1	82 (98.8)	188 (72.9)	270 (78.0)
DENV-2	1 (1.2)	36 (13.9)	37 (10.7)
DENV-3	0	2 (0.8)	2 (0.6)
DENV-4	0	31 (12.0)	31 (9.0)
DENV-1 and DENV-2	0	1 (0.4)	1 (0.3)
Missing	5	0	5

*Participants 0.5–30 years of age in 2012 and 0.5–20 years of age in 2013. DENV, dengue virus.

Screened participants had a mean age (+ SD) of 11.7 (+ 7.9; median 10; interquartile range 6–16); 6,207 (77.9%) were schoolchildren, university students, or nonschooled children. The main sources of household income were planting crops (61.0%), working in a factory (14.3%), and keeping a shop (13.4%). Participants reported low use of protective measures against mosquitoes, including mosquito coils in 787 (26.3%) households, insecticide sprays in 557 (18.6%) households, and larvicidal temephos in 374 (12.5%) households. Our investigation found uncovered water jars in 1,867 (62.7%) households and mosquito larvae in water containers of 1,663 (55.7%) households (Table 2).

**Table 2:** Participant and household characteristics with unadjusted and adjusted attack rate ratios for factors potentially associated with dengue virus infection, Cambodia, 2012–2013*

Characteristics	Infected	Uninfected	Total	Unadjusted ARR (95% CI)	Adjusted ARR (95% CI)
Participants	346	7,614	7,960		
Sex					
M	171	4,103	4,272	Referent	Referent
F	175	3,511	3,686	1.14 (0.92–1.40)	1.01 (0.82–1.24)
Age, y†					
0.5–<1	9 (2.6)	150 (2.0)	159 (2.0)	3.47 (1.65–7.32)	3.53 (1.67–7.46)
1–<5	108 (31.2)	1,701 (22.3)	1,809 (22.7)	3.98 (2.69–5.90)	4.04 (2.72–5.98)
5–<10	126 (36.4)	2,083 (27.4)	2,209 (27.8)	3.79 (2.56–5.60)	3.83 (2.59–5.67)
10–<15	71 (20.5)	1,675 (22.0)	1,746 (21.9)	2.59 (1.70–3.94)	2.55 (1.67–3.88)
15–30	32 (9.3)	2,005 (26.3)	2,037 (25.6)	Referent	Referent
Mean (+ SD, median)	8.5 (+ 5.7, 7)	11.9 (+ 8.0, 10)	11.7 (+ 7.9, 10)	–	–
Occupation‡					
Student, school or university	171 (49.8)	3,588 (47.2)	3,759 (47.3)	2.14 (1.35–3.41)	2.14 (1.34–3.41)
Preschool or unschooled	149 (43.2)	2,299 (30.2)	2,448 (30.8)	2.84 (1.79–4.54)	2.84 (1.78–4.54)
Planting crops	20 (5.8)	910 (12.0)	930 (11.7)	Referent	Referent
Other	5 (1.5)	809 (10.6)	814 (10.2)	0.28 (0.10–7.76)	0.28 (0.10–7.76)
Missing	1 (0.2)	8 (0.1)	9 (1.1)		
Relationship to index case-patient§					
Neighbor	260 (75.4)	6,309 (83.0)	6,569 (82.6)	Referent	Referent
Cousin	58 (16.8)	991 (13.0)	1,049 (13.2)	1.38 (1.01–1.89)	1.40 (1.02–1.90)
Sibling	23 (6.7)	251 (3.3)	274 (3.5)	2.11 (1.33–3.34)	2.24 (1.42–3.53)
Other	5 (1.2)	55 (0.7)	59 (0.7)	1.66 (0.59–4.65)	1.76 (0.34–4.90)
Missing	1 (0.2)	8 (0.1)	9 (1.1)		
Households	292¶	2,706	2,988		
Water source#					
Nonpiped	108 (36.3)	1,186 (43.7)	1,284 (43.0)	Referent	Referent
Piped	184 (63.7)	1,520 (56.3)	1,704 (57.0)	1.32 (1.03–1.69)	1.35 (1.06–1.71)
Primary source of income**					
Planting crops	176 (60.9)	1,648 (61.0)	1,824 (61.0)	Referent	Referent
Working in a factory	42 (14.5)	384 (14.2)	426 (14.3)	1.16 (0.84–1.62)	1.20 (0.87–1.66)
Shopkeeping	37 (12.8)	362 (13.4)	399 (13.4)	0.97 (0.67–1.40)	1.03 (0.72–1.48)
Fishing, farming, animal husbandry	14 (4.8)	55 (2.0)	69 (2.3)	1.98 (1.15–3.43)	2.02 (1.18–3.45)
Working in government	5 (1.7)	57 (2.1)	62 (2.1)	0.94 (0.38–2.30)	0.99 (0.41–2.37)
Other	15 (5.2)	193 (7.2)	208 (7.0)	0.76 (0.43–1.32)	0.85 (0.50–1.46)
Mosquito control measures††					
Temephos	26 (9.0)	348 (12.9)	374 (12.5)	0.70 (0.47–1.06)	0.73 (0.48–1.10)
Larvivorous fish	26 (9.0)	214 (7.9)	240 (8.3)	1.14 (0.75–1.74)	1.18 (0.78–1.79)
Treated mosquito netting	27 (9.3)	311 (11.5)	338 (11.3)	0.78 (0.52–1.17)	0.82 (0.55–1.21)
Treated jar cover	3 (1.0)	47 (1.7)	50 (1.7)	0.73 (0.24–2.24)	0.77 (0.26–2.27)
Coils	77 (26.6)	710 (26.3)	787 (26.3)	1.08 (0.82–1.41)	1.16 (0.89–1.51)
Insecticide spray	44 (15.2)	513 (19.0)	557 (18.6)	0.79 (0.57–1.10)	0.88 (0.63–1.22)
Environmental factors**					
Vegetable garden	57 (9.7)	546 (20.2)	603 (20.2)	0.89 (0.66–1.21)	0.89 (0.66–1.20)
Water collection around house	126 (43.6)	1,180 (43.7)	1,306 (44.7)	0.91 (0.71–1.15)	0.88 (0.70–1.12)
Uncovered water jars	178 (61.6)	1,689 (62.6)	1,867 (62.5)	0.96 (0.75–1.22)	0.97 (0.76–1.23)
Larvae in water containers	168 (58.1)	1,495 (55.4)	1,663 (55.7)	1.09 (0.86–1.39)	1.07 (0.85–1.37)
Distance from house to nearest water jar, m (+ SD)	1.5 (+ 2.2)	1.3 (+ 2.0)	1.3 (+ 2.0)	1.00 (0.98–1.02)	1.00 (0.98–1.02)
Missing for all items	3	7	10		

*Values are no. or no. (%) except as indicated. ARR, attack rate ratio; DENV, dengue virus. †Participants 0.5–30 years of age in 2012 and 0.5–20 years of age in 2013.  ‡Adjusted for sex. §Adjusted for age. ¶No. housesholds with >1 DENV case. #Nonpiped water comes from a river, pond, lake, or a well that does not have a pump; piped water comes from indoor or outdoor taps with a tube well and pump.  **Adjusted for sex and occupation. ††Adjusted for age, relationship to index case-patient, occupation, and primary source of income.

Among DENV-positive cases, boys and girls were equally affected at a mean (+ SD) age of 8.5 (± 5.7) years. Compared with persons 15–30 years of age, we found that children 1–10 years of age had a higher ARR of DENV infection (ARR 4.04 [95% CI 2.72–5.98] for those 1–5 years of age and ARR 3.83 [95% CI 2.59–5.67] for those 6–10 years of age). Siblings and cousins of index case-patients were more prone to DENV infection than neighbors were; siblings were 2.24 (95% CI 1.42–3.53) times and cousins 1.40 (95% CI 1.02–1.90) times more at risk for infection than neighbors. Participants who used piped water had a higher risk for DENV infection (ARR 1.35 [95% CI 1.06–1.71]) than did those who used nonpiped water. Households in which the main source of income was fishing, farming, or animal husbandry also had higher risks for infection (ARR 2.02 [95% CI 1.18–3.45]). Households reporting mosquito control–related parameters did not have a lower risk for DENV infection (Table 2). 

The main source of income was similarly distributed between households with ≥1 case and households with no cases (p = 0.272). Our multilevel model showed a notable clustering effect at the household level after adjustment (intraclass correlation coefficient 40.8%).

We found 26 (7.5%) case-patients, 17 (65.4%) male and 9 (34.6%) female, who were positive for DENV infection but remained asymptomatic. We found serotypes DENV-1, DENV-2, and DENV-4 in our study group (Table 3). We used a multilevel approach to explore the role of specific serotypes and participant-level factors, such as age, gender, and relationship to the index case-patient, a proxy for common genetic background, with being DENV-positive and asymptomatic. We found that only family relationship to the index case-patient was associated with asymptomatic infection. We did not identify a cluster effect or associated factors.

**Table 3:** Univariate tests for associations between sociodemographic factors and infecting serotypes with asymptomatic dengue virus infections, Cambodia, 2012–2013*

Factor	Asymptomatic, n = 26	Symptomatic, n = 320	p value†
Sex			
M	17	154	0.09
F	9	166	
Age, y			
0.5 to <1	0	9 (2.8)	0.976
1–5	9 (34.6)	99 (30.9)	
6–10	9 (34.6)	117 (36.6)	
11–14	5 (19.2)	66 (20.6)	
15–30	3 (11.5)	29 (9.1)	
Mean (+ SD, median)	9.2 (+ 7.2, 8)	11.0 (+ 7.1, 10)	
Relationship to index case-patient			
Neighbor	17 (65.4)	243 (76.0)	0.004
Cousin	5 (19.2)	53 (16.6)	
Sibling	1 (3.9)	22 (6.9)	
Other	3 (11.5)	1 (0.3)	
Source of household income			
Planting crops	14 (53.8)	192 (60.0)	0.812
Working in a factory	3 (11.5)	53 (16.6)	
Shopkeeping	5 (19.2)	36 (11.3)	
Fishing, farming, animal husbandry	1 (3.9)	17 (5.3)	
Working in government	0	6 (1.9)	
Other or missing	3 (11.5)	16 (5.0)	
DENV serotype‡			
DENV-1	21 (80.8)	249 (79.1)	0.892
DENV-2	2 (7.7)	35 (11.1)	
DENV-3	0	2 (0.6)	
DENV-4	3 (11.5)	28 (8.9)	

*Values are no. (%) patients except as indicated. DENV, dengue virus. †By Fisher test or χ^2^ test.  ‡Data for 5 symptomatic patients were missing, and another patient was excluded from analysis because of co-infection with DENV-1 and DENV-2.

## Discussion

We screened 7,960 participants in communities in Cambodia during 2012 and 2013 and found 346 (4.3%) participants infected by DENV; 26 (7.5%) remained asymptomatic before, during, and after our study. We found comparable attack rates, 37/1,000 persons in 2012 and 46/1,000 persons in 2013, to other community investigations conducted in Cambodia. For instance, another study reported DENV attack rates of 13.4–57.8 cases/1,000 persons during 2006–2008 (*4*). Previous studies only included participants ≤20 years of age, but we included persons 0.5–30 years of age with confirmed DENV infection, even symptomatic but afebrile case-patients, who were 31.8% of the DENV infections in this study. We noted that attack rates were lower in PI-V, 14.7/1,000 participants (14/952), than in PI-H, 47.4/1,000 participants (332/7,008). Circulation of DENV around febrile index case-patients identified through PI-V was less intense, but with more asymptomatic cases, than around index case-patients identified through PI-H. Aside from possible detection biases (*17*), multiple factors could explain this observation and deserve further research.

Our study documented cases of DENV infection in transmission clusters located around index case-patients. We found that 26.6% of DENV-confirmed case-patients reported clinical symptoms, including headache and muscle pain, but no fever even in the absence of antipyretics, comparable to data from Thailand, where 40.4% of the DENV cases remained afebrile (*17*). The appearance of afebrile DENV-infected patients raises potential concerns for case definitions for detection, especially of imported cases in at-risk countries.

An additional 7.5% of DENV-confirmed case-patients had no symptoms during the 10-day course of clinical monitoring, a considerably lower rate than estimates from other prospective studies (*5*,*18*–*21*). Published sources refer to inapparent infections, often defined as afebrile clinical complaints with biologic evidence of DENV infection, ranging from 20% to 80% of cases (*19*,*22*,*23*). Previous studies used different definitions of asymptomatic infection than ours, but the major difference lies in follow-up monitoring. Other retrospective studies used school or work absenteeism as a basis for follow-up (*19*). Strictly asymptomatic patients, such as those we describe, escape detection by surveillance or control measures, infect mosquitoes, and might disproportionately contribute to DENV transmission (*5*).

The DENV burden documented through hospital-based surveillance of febrile case-patients in Thailand and Vietnam showed a shift to older age groups (*24*,*25*). Our active, systematic case-finding system to identify DENV in villages in Cambodia found the attack rate was highest in children <10 years of age, which is what we expected in a dengue-endemic country with frequent outbreaks demonstrated in other careful studies (*26*). This finding raises concerns because recommendations for the only licensed dengue vaccine are for use in persons 9–45 years of age with demonstrated evidence of past DENV infection (*27*). Our study demonstrates that children in rural Cambodia might have undergone >1 DENV infection before 9 years of age, reducing the potential cost-effectiveness of vaccination.

Few studies have explored the role of socioeconomic status, which might be a proxy for peridomestic environmental management, on DENV infection in Southeast Asia. Often, the direction of the association is unclear and socioeconomic status has entirely different associations depending on the setting (*28*). Our study shows that the adjusted risk for DENV infection was highest in households in which the main source of income was from fishing, farming, or animal husbandry, activities associated with lower average household income in Cambodia.

We found temephos provided no additional protection against DENV infection after adjusting for other factors. Although temephos is effective in reducing *Aedes* spp. larval populations in water storage jars, its use did not correlate with lower DENV transmission in Cambodia or elsewhere (*8*), due to incorrect distribution coverage, dosage, and placement (*29*) or multiple vector breeding sites. In addition, increases in temephos-resistant *A. aegypti* mosquito larvae have been documented in Cambodia (*30*).

Unexpectedly, we found a higher risk for DENV with piped water as a main water source after adjusting for other factors, contrary to a study in Thailand (*6*). However, piped water with suboptimal sanitation in Cambodia might contribute to collection of water in or around households that could become breeding sites for DENV-transmitting mosquitoes.

We found that 40.8% of the variability in probability of being DENV infected was explained by differences between households. Those living in the same household as an index case-patient were 2.11 times more likely to be infected, consistent with other published sources. A study in Mexico found that the risk for infection for those living with an index case-patient was twice that of someone living in a 50-meter radius of an index case-patient (*31*). This relationship was further described in a cluster study in Thailand that showed decreased risk for infection with increasing distance from the index case household (*31*). This clustering effect around an index case, however, seems to occur only on a short temporal scale, at least in urban settings (*32*).

Rates and severity of illness after infection with the different DENV serotypes differ widely (*33*,*34*). The only notable epidemiologic factor associated with asymptomatic DENV infections in our study was being family-related to the index case-patient. Another study showed that adaptive immune responses against DENV differ between persons with symptomatic and asymptomatic DENV infection (*35*), which might explain our observations. We found no other associated epidemiologic factor, including age or cluster effects. Although the ratio of male to female participants was twice as high among asymptomatic than symptomatic participants, this finding was not statistically significant, likely due to sample size. 

Although the size and duration of our study confer strength to our data, it might suffer from biases and limitations, especially due to the small number of strictly asymptomatic DENV-positive participants after stratifying by DENV serotype. We found dengue incidence rates highest in young children. These data might be biased because we focused on investigating clusters around an index case, perhaps overestimating the incidence in the general population. DENV circulation, however, is intense in children in Cambodia, and these figures remain comparable to those found in dengue studies that use different methods, ranging from 20 to 80 per 1,000 person-seasons (*1*). Furthermore, we did not capture cases referred to the private sector, lowering our estimates somewhat. Healthy male workers often were away at the time of the investigations, possibly leading to an overestimation of DENV incidence. These workers, however, are >18 years of age, but DENV infections occur mainly in persons <15 years of age in Cambodia (*4*).

Documentation bias might also have pulled our risk factor estimates toward the null. We did not document solid waste disposal in our study, but comparatively high Breteau index values have been reported in Cambodia (*29*). In addition, we could have missed details or misrepresented implementation of mosquito-control measures. Despite the potential misclassification, mosquito-control measures remain nondifferential and likely had no major effect on our risk estimates.

Further, 7.5% of our DENV-infected participants remained strictly asymptomatic. Aside from case definition issues we discuss, our method of screening for DENV around symptomatic cases might have underestimated the number of asymptomatic DENV infections. In addition, we did not enroll persons who tested negative for DENV IgM, NS1-capture ELISA, and qRT-PCR. Some of these persons might have been infected but not yet mounted an IgM response, so that NS1 and viral RNA titers had already receded to undetectable levels when we tested them. This strict case definition might have underestimated the incidence of asymptomatic cases, but a precise retrospective documentation of such cases would be extremely difficult. Similarly, we retrospectively conducted MAC-ELISA on samples collected during perifocal investigations and identified 11 cases of IgM seroconversion in the absence of PCR- or NS1-positive tests. Even in the context of JEV cocirculation, some of these cases could have been true DENV infections, but including them would not have changed the overall estimated attack rate. Previous studies suggested virus serotype might affect severity and types of symptoms and observed that DENV-1 infections more frequently were associated with clinically apparent illness (*36*,*37*). Virus molecular analysis studies are ongoing to determine whether specific strains cause more asymptomatic infection than others. Furthermore, DENV infection in Cambodia occurs mainly in children who might be more likely to answer positively to daily-repeated questions on dengue symptoms, somewhat underestimating asymptomatic cases. Having implemented careful and thorough 10-day clinical assessment of objective symptoms in each asymptomatic DENV-positive participant, we believe these figures reflect the true proportion of strictly asymptomatic DENV infection in our setting. However, we collected our findings mainly in children with DENV-1 infection in Cambodia. Whether these findings are directly applicable to other epidemiologic settings, populations, or virus serotypes or genotypes remains to be determined (*33*).

Finally, vaccination against JEV might have led to cross-protection against symptomatic dengue. Data on JEV vaccination were not collected during perifocal investigations. According to local health centers, however, JEV vaccine has been provided only recently and only for children 9–24 months of age. In our study, only 3 children were DENV-positive in that age category.

Our study demonstrates that systematically relying on fever for DENV case definition can underestimate cases and hinder control efforts in areas with potential vectors and at risk for DENV introduction. We found 7.5% of DENV-infected participants remained strictly asymptomatic, which has wide-ranging epidemiologic consequences. Undetected sources can increase transmission (*5*), a factor that must be taken into account in future vaccine coverage and vaccine effectiveness studies. The attack rate differences observed around febrile index case-patients detected in village surveillance and index case-patients detected in hospital surveillance deserve further study. In-depth virus (*36*) and human genetic studies could contribute useful insights (*33*,*35*). Our strict definition of asymptomatic DENV infections should be considered when designing studies that aim to elucidate the pathophysiological mechanisms of dengue disease.
